# The Relationship between COVID-19 and Hypothalamic–Pituitary–Adrenal Axis: A Large Spectrum from Glucocorticoid Insufficiency to Excess—The CAPISCO International Expert Panel

**DOI:** 10.3390/ijms23137326

**Published:** 2022-06-30

**Authors:** Mojca Jensterle, Rok Herman, Andrej Janež, Wael Al Mahmeed, Khalid Al-Rasadi, Kamila Al-Alawi, Maciej Banach, Yajnavalka Banerjee, Antonio Ceriello, Mustafa Cesur, Francesco Cosentino, Massimo Galia, Su-Yen Goh, Sanjay Kalra, Peter Kempler, Nader Lessan, Paulo Lotufo, Nikolaos Papanas, Ali A. Rizvi, Raul D. Santos, Anca P. Stoian, Peter P. Toth, Vijay Viswanathan, Manfredi Rizzo

**Affiliations:** 1 Department of Endocrinology, Diabetes and Metabolic Diseases, University Medical Center Ljubljana, 1000 Ljubljana, Slovenia; mojcajensterle@yahoo.com (M.J.); rokherman2@gmail.com (R.H.); 2 Department of Internal Medicine, Faculty of Medicine, University of Ljubljana, 1000 Ljubljana, Slovenia; 3 Heart and Vascular Institute, Cleveland Clinic, Abu Dhabi P.O. Box 112412, United Arab Emirates; wmahmeed@gmail.com; 4 Medical Research Center, Sultan Qaboos University, Muscat 113, Oman; khalid77@squ.edu.om; 5 Department of Training and Studies, Royal Hospital, Ministry of Health, Muscat 113, Oman; alalawikamila@gmail.com; 6 Department of Preventive Cardiology and Lipidology, Medical University of Lodz (MUL), 90-419 Lodz, Poland; maciej.banach@umed.lodz.pl; 7 Polish Mother’s Memorial Hospital Research Institute (PMMHRI), 93-338 Lodz, Poland; 8 Cardiovascular Research Centre, University of Zielona Gora, 65-417 Zielona Gora, Poland; 9 Department of Biochemistry, Mohamed Bin Rashid University, Dubai P.O. Box 505055, United Arab Emirates; yajnavalka.banerjee@mbru.ac.ae; 10 IRCCS MultiMedica, 20099 Milan, Italy; antonio.ceriello@hotmail.it; 11 Clinic of Endocrinology, Ankara Güven Hospital, 06540 Ankara, Turkey; drcesur@yahoo.com; 12 Unit of Cardiology, Karolinska Institute and Karolinska University Hospital, University of Stockholm, 171 77 Stockholm, Sweden; francesco.cosentino@ki.se; 13 Department of Biomedicine, Neurosciences and Advanced Diagnostics (Bind), University of Palermo, 90127 Palermo, Italy; massimo.galia@you.unipa.it; 14 Department of Endocrinology, Singapore General Hospital, Singapore 169856, Singapore; goh.su.yen@singhealth.com.sg; 15 Department of Endocrinology, Bharti Hospital & BRIDE, Karnal 132001, India; brideknl@gmail.com; 16 Department of Medicine and Oncology, Semmelweis University, 1085 Budapest, Hungary; kempler.peter@med.semmelweis-univ.hu; 17 The Research Institute, Imperial College London Diabetes Centre, Abu Dhabi P.O. Box 48338, United Arab Emirates; nlessan@icldc.ae; 18 Center for Clinical and Epidemiological Research, University Hospital, University of São Paulo, São Paulo 05403-000, Brazil; paulo.lotufo@gmail.com; 19 Diabetes Center, Second Department of Internal Medicine, Democritus University of Thrace, University Hospital of Alexandroupolis, 68100 Alexandroupoli, Greece; papanasnikos@yahoo.gr; 20 Department of Medicine, University of Central Florida College of Medicine, Orlando, FL 32827, USA; ali.rizvi@ucf.edu; 21 The Heart Institute (InCor), University of Sao Paulo Medical School Hospital, São Paulo 05403-000, Brazil; rauldsf@gmail.com; 22 Hospital Israelita Albert Einstein, São Paulo 05652-900, Brazil; 23 Faculty of Medicine, Diabetes, Nutrition and Metabolic Diseases, Carol Davila University, 050474 Bucharest, Romania; ancastoian@yahoo.com; 24 Cicarrone Center for the Prevention of Cardiovascular Disease, The Johns Hopkins University School of Medicine, Baltimore, MD 21205, USA; peterptoth59.com@gmail.com; 25 Diabetes Research Centre, Chennai 600013, India; drvijay@mvdiabetes.com; 26 Department of Health Promotion, Mother and Child Care, Internal Medicine and Medical Specialties (Promise), University of Palermo, 90127 Palermo, Italy; manfredi.rizzo@unipa.it

**Keywords:** SARS-CoV-2, glucocorticoids, hypothalamic–pituitary–adrenal axis, hypercortisolism, adrenal insufficiency

## Abstract

Coronavirus disease 2019 (COVID-19) is a highly heterogeneous disease regarding severity, vulnerability to infection due to comorbidities, and treatment approaches. The hypothalamic–pituitary–adrenal (HPA) axis has been identified as one of the most critical endocrine targets of severe acute respiratory syndrome coronavirus 2 (SARS-CoV-2) that might significantly impact outcomes after infection. Herein we review the rationale for glucocorticoid use in the setting of COVID-19 and emphasize the need to have a low index of suspicion for glucocorticoid-induced adrenal insufficiency, adjusting for the glucocorticoid formulation used, dose, treatment duration, and underlying health problems. We also address several additional mechanisms that may cause HPA axis dysfunction, including critical illness-related corticosteroid insufficiency, the direct cytopathic impacts of SARS-CoV-2 infection on the adrenals, pituitary, and hypothalamus, immune-mediated inflammations, small vessel vasculitis, microthrombotic events, the resistance of cortisol receptors, and impaired post-receptor signaling, as well as the dissociation of ACTH and cortisol regulation. We also discuss the increased risk of infection and more severe illness in COVID-19 patients with pre-existing disorders of the HPA axis, from insufficiency to excess. These insights into the complex regulation of the HPA axis reveal how well the body performs in its adaptive survival mechanism during a severe infection, such as SARS-CoV-2, and how many parameters might disbalance the outcomes of this adaptation.

## 1. Introduction

COVID-19 is a highly heterogeneous disease regarding disease severity, vulnerability to infection due to underlying comorbidities, and administered medications. It has been demonstrated to be a systemic disease with effects extending beyond the respiratory system and often presents with protracted clinical manifestations lasting beyond the initial infection period [1,2]. The ubiquitous expression of angiotensin-converting enzyme 2 (ACE2), the primary receptor responsible for the entry of SARS-CoV-2 at the cellular level, combined with widespread endothelial damage and altered immune response, partially explain the multisystemic presentation of COVID-19 [1]. The involvement of the endocrine system in COVID-19 is so relevant that an “endocrine phenotype” of COVID-19 has progressively acquired clinical relevance [1]. However, while the contribution of endocrine dysfunction to the severity and outcomes of COVID-19 remains to be fully elucidated, the impact of SARS-CoV-2 on the endocrine system may be severely underreported due to the lack of awareness of the public and clinicians [2,3]. In particular, adrenal glands, with a crucial role in priming the immune system, might be a vulnerable and vital target during COVID-19, with direct and indirect effects on the overall outcomes. Furthermore, systemic corticosteroids remain the leading treatment choice in moderate and severe COVID-19 cases with acute and persistent effects on the hypothalamic–pituitary–adrenal (HPA) axis. In addition, pre-existing adrenal disorders may impact the susceptibility and severity of COVID-19, and special care is needed in the management of these patients during the current pandemic. Finally, with the long-term impact of COVID-19 presenting an increasing challenge for health care systems worldwide, the extent of the HPA axis’s contribution to this problem as a likely endocrine culprit remains a topic for future research.

This article will comprehensively review the relationship between SARS-CoV-2 infection and the HPA axis. We will cover topics from the rationale for glucocorticoid use in COVID-19 patients, and potential direct and indirect mechanisms that might cause the dysfunction of the HPA axis during SARS-CoV-2 infection to whether patients infected with COVID-19 are in higher-risk categories for developing more severe illness modalities due to endogenous- or exogenous-induced hypo or hypercortisolism.

## 2. Glucocorticoids in the Management of COVID-19 Patients

Due to the rapid onset of the current pandemic, frontline clinicians were required to undertake treatment decisions despite vast gaps in the knowledge about the novel coronavirus, its clinical course, and only weak evidence from the first reports regarding the efficacy of initially proposed agents [4]. The lack of effective antiviral agents quickly shifted the focus towards the more known complications associated with COVID-19, such as acute respiratory distress syndrome (ARDS) and cytokine storm syndrome. Clear evidence that the cytokine deregulation and excessive inflammatory response might be more detrimental than uncontrolled viral replication in critical COVID-19 cases raised the possibility of using antiinflammatory agents in an effort to control and resolve the illness [5].

Glucocorticoids have been widely used in the previous severe acute respiratory syndrome (SARS) and the Middle East respiratory syndrome (MERS) epidemics and ARDS in general; however, the data regarding their efficacy were contradictory and generally of low quality [6]. They exert potent antiinflammatory effects across both innate and adaptive immune systems through the DNA-dependent regulation of antiinflammatory proteins, non-genomic modulation of inflammation, and direct protein interference of transcription factors such as nuclear factor kappa B [7,8,9]. In early 2020, a retrospective study of 201 patients admitted with confirmed COVID-19 pneumonia in Wuhan, China, provided the first evidence supporting their usage by demonstrating that treatment with methylprednisolone was associated with a reduced risk of death among patients with ARDS [10]. However, in the early stages of the pandemic, medical professionals were apprehensive about the use of immunosuppressants in the treatment of infectious disease, and previous evidence of the possibility of prolonged viral shedding and bacterial superinfection with their usage added to the concern [7]. In addition, due to the ubiquitous glucocorticoid receptor (GR) expression and large-scale GR-mediated effects, there are well-documented and numerous off-target effects related to glucocorticoid treatment, mainly dependent on its dose and duration [7]. The spectrum of abnormalities can range from initial symptoms of hypercortisolism in patients with individual hypersensitivity to prolonged HPA axis suppression after drug withdrawal [6]. Therefore, in January 2020, the World Health Organization (WHO) issued provisional guidelines against the routine use of glucocorticoids in the management of patients with COVID-19 [11]. Many subsequent guidelines from other groups also stated that glucocorticoids were either contraindicated or not recommended.

Multiple large-scale randomized control trials (RCTs) began assessing glucocorticoid efficiency, most notably the RECOVERY trial in the United Kingdom. Only about 100 days after the study protocol was drafted, on June 16, 2020, the study showed that dexamethasone is effective in reducing 28-day mortality among patients who were receiving either invasive mechanical ventilation (26.2% vs. 29.3%) or oxygen alone (23.3% vs. 26.2%), but not among those receiving no respiratory support (17.8% vs. 14.0%) [12]. The results immediately changed the hospital management guidelines across the globe, and most other trials suspended enrolment. In September 2020, the WHO provided a strong recommendation for systemic corticosteroids in patients with severe and critical COVID-19 and a weak or conditional recommendation against systemic corticosteroids in patients with non-severe disease [13]. Those guidelines were further supported by a high level of evidence for glucocorticoid effectiveness in hospitalized patients who need respiratory support in a prospective meta-analysis of seven RCTs by the WHO Rapid Evidence Appraisal for COVID-19 Therapies Working Group, which concluded that the initiation of systemic glucocorticoid was associated with lower 28-day all-cause mortality in critically ill patients with COVID-19. Included trials evaluated daily glucocorticoid equivalent doses ranging from 6 mg to 29 mg of dexamethasone; however, the analysis was not powered to assess the optimal agent, dose, or treatment duration [14]. 

Systemic glucocorticoids continue to be recommended for moderately or severely ill patients in both the WHO guidance for the clinical management of COVID-19 and the National Institutes of Health (NIH) COVID-19 guidelines [13,15]. Although the WHO guidance does not recommend a particular glucocorticoid dose, it recommends regimens lasting 7–10 days [13]. The NIH guidelines recommend 6 mg of dexamethasone once daily for ten days or until hospital discharge [15]. Many potential beneficial effects of glucocorticoids in the treatment of COVID-19 have been proposed. However, whether they are successful due to the general supportive benefits of glucocorticoids in critically ill patients, mitigation of the cytokine release syndrome and ARDS [12,16], the treatment of undiagnosed adrenal insufficiency (AI) [17,18], or the suppression of endogenous cortisol secretion with reduced effects on mineralocorticoid receptors [19] has yet to be clarified [20].

Despite considerable efforts, it is challenging to derive definitive conclusions on an optimal glucocorticoid agent, or its timing, dose, and duration from the current literature [8]. Mechanistically methylprednisolone achieves higher lung-to-plasma ratios in animal models than dexamethasone, and smaller trials have demonstrated its superiority to dexamethasone; however, the therapeutic dosing was not equivalent between groups [21,22]. Regarding the best possible initiation time for the treatment, the current evidence suggests their usage in the second week of infection, when inflammatory instead of infectious mechanisms predominate [8,23,24]. In trials so far, different dosage regimes have been reported in patients with severe COVID-19, from consistent doses throughout the treatment course, to the administration of a tapering dose, or the administration of pulse doses for a short period [8]. 

The amount and duration of glucocorticoid administration also need to be carefully adjusted because a series of side effects may occur after long-term or high-dose use [25]. In a recent meta-analysis, glucocorticoid treatment was significantly associated with an increased risk of viral clearance delay in COVID-19 patients receiving high or medium doses compared to those taking low doses [26]. Lastly, the ability of synthetic glucocorticoids to induce a persistent inhibition of the HPA axis, even after a short period of treatment, is not clearly predictable because the inter-individual pharmacokinetic differences, sensitivity variations in GRs, pathophysiological changes in cortisol dynamics in acute illness, and the specific guidelines regarding dose tapering are lacking [27].

## 3. Glucocorticoid-Induced Adrenal Insufficiency in COVID-19 Patients

Systemic glucocorticoids exert negative feedback on corticotrophin-releasing hormone (CRH) producing neurons and pituitary corticotroph cells. This leads to reduced adrenal cortisol production and, after prolonged exposure, adrenal cortical hypoplasia and atrophy [28]. After the effect of glucocorticoid therapy on the HPA axis has worn off, the production of adrenocorticotropic hormone (ACTH) and CRH typically recover first, followed by that of cortisol, which can remain suppressed in the long term if adrenal atrophy has ensued [28].

The risk of developing glucocorticoid-induced adrenal insufficiency (GI-AI) is challenging to predict on an individual basis. The glucocorticoid formulation, dose, treatment duration, and underlying health problems can affect this risk, but a great degree of overlap exists [28,29]. In the general population, only 2% of patients reported symptoms consistent with GI-AI, yet several cases of adrenal crisis and hospital admission for GI-AI have been described, including a few deaths [28]. 

Currently, the monitoring of GI-AI in COVID-19 patients is unsystematic, sporadic, and reliant on individual clinicians’ knowledge and awareness. Patients with recently resolved COVID-19 and untreated GI-AI might present with nonspecific signs and symptoms such as chronic fatigue, difficulty thinking or concentrating, musculoskeletal pain, insomnia, mood changes, and low blood pressure that can be attributed to other causes, including long COVID symptoms. The actual risk of GI-AI in post-COVID-19 patients has not been assessed in a larger cohort. 

Many endocrinologists have helped develop national and local recommendations from a patient safety and cost-effectiveness perspective, including evidence-based guidelines for testing, treating, and monitoring for GI-AI in the general population. We now need to provide effective, uniform, and economical monitoring by analyzing these different regional and national protocols. Meanwhile, as recommended for glucocorticoid users in the general population [29,30], we need a low threshold to replace and test for GI-AI after glucocorticoid withdrawal while we wait for more evidence in COVID-19 patients. This is particularly important for patients with nonspecific symptoms after cessation, including those with symptoms attributed to long COVID.

## 4. The Impact of COVID-19 Infection on Hypothalamic–Pituitary–Adrenal Axis

**Figure 1:**
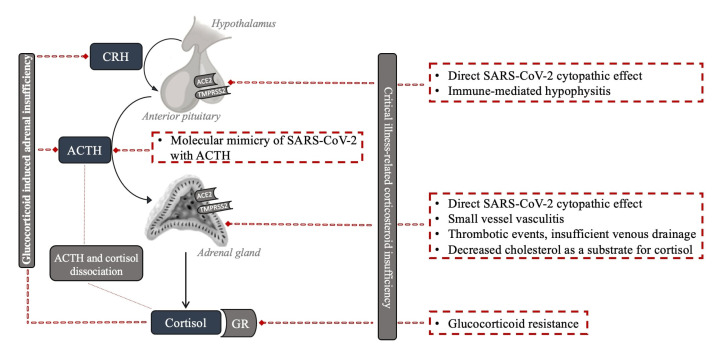
Potential mechanisms of HPA axis impairment with SARS-CoV-2 infection.

Beyond GI-AI, several additional mechanisms can cause the observed impact of SARS-CoV-2 infection on the HPA axis. The various potential mechanisms by which SARS-CoV-2 can impair the HPA axis are presented in Figure 1.

### 4.1. Critical Illness-Related Corticosteroid Insufficiency

Firstly, deregulation of the HPA axis may be encountered as part of the development of functional AI, as has been described in critical illness from 2000 onwards based on the initial work by Annane et al. in septic shock [31], later named critical illness-related corticosteroid insufficiency (CIRCI) [32]. This functional AI is hypothesized to be mainly due to inadequate cortisol secretion for the modulation of inflammatory responses in critically ill patients. Multiple mechanisms, including (i) reduced albumin or cortisol-binding globulins (CBGs), (ii) decreased affinity, (iii) reduced number of adrenal gland cortisol receptors, (iv) or increased activity of 11-β hydroxysteroid dehydrogenase type 2 resulting in cortisol inactivation, have been proposed in this regard [33]. In addition, higher levels of cytokines observed in patients with CIRCI could directly suppress ACTH release during critical illness because the pituitary gland is not protected by the blood–brain barrier [6]. New research performed over the last 10 years has led to the insight that the term CIRCI should be explicitly used for a condition that may develop in prolonged critically ill patients that are at risk of acquiring central adrenal insufficiency. In that setting, the adrenal cortex, depleted from ACTH-mediated trophic signaling for a prolonged period, may become structurally and functionally impaired, resulting in insufficient cortisol production [34]. Regardless of the potential mechanisms, severe COVID-19 cases are at potential risk of developing CIRCI. A small study reported CIRCI diagnosis in six out of nine critically ill patients with COVID-19 [17].

### 4.2. Adrenal Gland

The expression of two crucial viral receptors through which SARS-CoV-2 enters host cells, ACE2 and transmembrane protease serine 2 (TMPRSS2), have been documented in the adrenal zona fasciculata and reticularis of the adrenal gland [17,35]. Autopsy studies showed adrenal histomorphologic alterations that could be assigned to the SARS-CoV-2 infection, implying a direct cytopathic effect [36,37]. The adrenals showed mainly acute fibrinoid necrosis of small vessels, primarily affecting arterioles in the adrenal parenchyma, capsule, and periadrenal adipose tissue [38]. Additional findings included subendothelial vacuolization and apoptotic debris without significant signs of inflammation, parenchymal infarctions, or thrombosis. In another autoptic study of fatal COVID-19 cases, the authors identified SARS-CoV-2 and its replication in the adrenal glands, which co-localized with ACE2 and TMPRSS2, mainly in epithelial but also in mesenchymal and endothelial cells [35,39,40]. Some reports specifically observed chronic inflammation with perivascular distribution and vasculitis of the small vessels. The autopsy studies have involved a very limited number of patients so far and only included those more severely affected by multi-organ failure. Whether the histomorphologic alterations led to altered cortisol dynamics or induced insufficiency is questionable. 

Furthermore, AI in COVID-19 patients may be due to thrombotic events [41]. Incidental adrenal computed tomography findings compatible with adrenal infarction have been reported in 23% of patients with severe COVID-19 [42], the vast majority with bilateral involvement. However, the clinically relevant cases are less frequently reported [43]. Of note, two out of nine clinically relevant cases had positive antiphospholipid antibodies prior to COVID-19 diagnosis, suggesting a previously described link between the antiphospholipid syndrome and the risk of bilateral adrenal infarction in non-COVID-19 patients as a potential mechanism [43,44,45,46].

The adrenal gland’s venous drainage might be another culprit [47]. The high stress from SARS-CoV-2 infection might be a limiting factor for a solitary suprarenal vein due to ACTH-induced arteriolar dilation, leading to vascular stasis and subsequent adrenal damage [48]. Moreover, decreased cholesterol, in the main form of high-density lipoprotein (HDL), as one of the main substrates for cortisol synthesis, might lead to hypocortisolism in severe COVID-19 infection [49].

### 4.3. Pituitary Gland and Hypothalamus

AI may also occur due to central dysfunction. An impaired adrenocortical response was reported in 28 patients with COVID-19 with plasma cortisol and ACTH concentrations indicating central hypocortisolism [18]. Several potential mechanisms have been hypothesized regarding the etiology of COVID-19-related central hypocortisolism. The expression of two receptors through which SARS-CoV-2 enters host cells, ACE2 and TMPRSS2, have also been documented in the hypothalamus and pituitary [50], making them possible direct cytopathic targets of SARS-CoV-2. In a postmortem study of COVID-19 patients, areas of necrosis/infarction were seen in one out of the nineteen pituitaries [51]. Genome sequences of SARS-CoV-2 have also been detected in the pituitary and hypothalamus postmortem studies, implying a direct hypothalamic injury induced by the virus [52,53]. Some authors also suggested a reversible immune-mediated hypophysitis [54].

A recent study in male mice established that the S1 subunit of the spike protein of SARS-CoV-2 crosses the blood–brain barrier and is taken up by brain regions that include the cortex, hypothalamus, and hippocampus, areas of particular importance for HPA axis control [55]. In small autoptic studies in patients infected with severe acute respiratory syndrome coronavirus 1 (SARS-CoV-1), the somatotroph, thyrotroph, and corticotroph pituitary cells’ number, as well as the respective hormone immunoreactivity, were reduced. However, the opposite was observed for mammotroph and gonadotroph pituitary cells [56]. Whether there are similar alterations with SARS-CoV-2 infection remains unknown. 

Furthermore, it has been hypothesized that SARS-CoV-1 inhibits the adrenal stress responses causing a relative adrenocortical insufficiency via molecular mimicry of specific sequences of SARS-CoV-1 with ACTH and the immune response that cross-reacts with ACTH [52,57]. However, currently, there is no evidence to support this intriguing hypothesis. Another possibility might be related to cytokines, as in CIRCI. Extensive cytokine production (interleukin (IL)-1, IL-6, TNF-α, monocyte chemoattractant protein 1 (MCP1), and granulocyte-colony stimulating factor (G-CSF)) during COVID-19 infection reduces ACTH release and decreases its effect on adrenal tissue [58,59,60,61].

### 4.4. Resistance to Cortisol Action at the Level of Glucocorticoid Receptor and Postreceptor Signaling

The immunological, metabolic, and hemodynamic actions of glucocorticoids are mediated by a ubiquitous intracellular receptor, the GR [62]. The GR–cortisol complex translocates from the cytosol to the nucleus, where it exerts transcriptional activity, resulting in the inhibition of the inflammatory response. Preclinical septic models have reported glucocorticoid resistance in critical illness [62]. Glucocorticoid resistance may be a consequence of decreased GR mRNA and protein expression, reduced GR affinity for the ligand and nuclear translocation, and/or impaired DNA binding. 

In patients with COVID-19, there is no evidence of resistance to cortisol action since the strong activation of an endogenous cortisol response to SARS-CoV-2 has been detected. It is, however, possible that the strong response is still insufficient to achieve maximum immunosuppression, providing grounds for corticotherapy in patients with COVID-19. In a recent study of single-cell RNA sequencing data, functionally active GR subunit mRNA expression from the bronchoalveolar lavage fluid was decreased in severe COVID-19 patients compared to mildly affected patients. The authors suggested that this might reflect a pathologic down-regulation of this endogenous immunomodulatory mechanism in patients with severe COVID-19 infection, which could be restored pharmacologically with corticosteroid therapy [63].

### 4.5. Dissociation between Cortisol and ACTH Regulation

Another phenomenon should be considered when interpreting the potential HPA axis deregulation in COVID-19 patients. ACTH and cortisol dissociation, attributed to the dependence of cortisol secretion from factors other than ACTH, has been described in severe illness. Cytokines, which are expected to be higher in the more severe illness, can stimulate cortisol secretion independently from ACTH. Reduced peripheral cortisol metabolism, resulting in increased systemic half-life, represents an additional ACTH-independent mechanism of increased cortisol levels with a consequent feedback reduction of ACTH [64]. In one study, ACTH levels tended to also be lower in those with moderate to severe COVID-19 with simultaneously increased cortisol levels, consistent with the published evidence of a dissociation between cortisol and ACTH levels in severe illness [65].

### 4.6. Clinical Data during Acute COVID-19 Infection

Beyond the above hypotheses, there are limited clinical data on HPA axis deregulation in COVID-19 patients during an acute infection. One limitation is the difficulty of performing a complete and exact evaluation of the HPA axis in patients with COVID-19 who are glucocorticoid dependent [66]. AI is a disease that is probably unrecognized in clinical settings during COVID-19. A systemic review of 10 studies that reported AI occurrence in patients who suffered from COVID-19 revealed the AI prevalence ranged from 3.1% to as high as 64.3%, indicating a high prevalence of AI in the COVID-19 pandemic without the clarification of the extent and type of AI [47]. Furthermore, some research groups evaluated the association of cortisol levels with the severity of COVID-19 disease, with conflicting outcomes.

In 403 non-critically ill COVID-19 patients, within 48 h of hospital admission, cortisol levels were significantly higher (619 (456–833) nmol/L) than in patients admitted to the hospital for other reasons (519 (378–684) nmol/L), indicating a marked and appropriate acute cortisol stress response (*p* < 0.0001) [67]. In a study of 144 COVID-19 critically ill patients, a multivariate logistic regression analysis demonstrated that mortality was associated with higher cortisol levels (odds ratio: 1.2; 95% confidence interval: 1.08–1.35; *p* = 0.001) and the cortisol cutoff point was 855 nmol/L for predicting mortality among COVID-19 patients [68]. A positive association with disease severity was also confirmed in a study that reported higher cortisol levels in COVID-19 patients with moderate to severe disease (433 (353–571) nmol/L) compared to those with mild disease (370 (279–454) nmol/L) (*p* = 0.053) [69]. A small study reported cortisol levels below 300 nmol/L (10 μg/dL) in a significant number of asymptomatic/mild cases, a finding that was not observed in repeated testing after a few days in most patients [18]. The researchers reported that doubling cortisol levels increased mortality by 42% [67].

**Figure 2:**
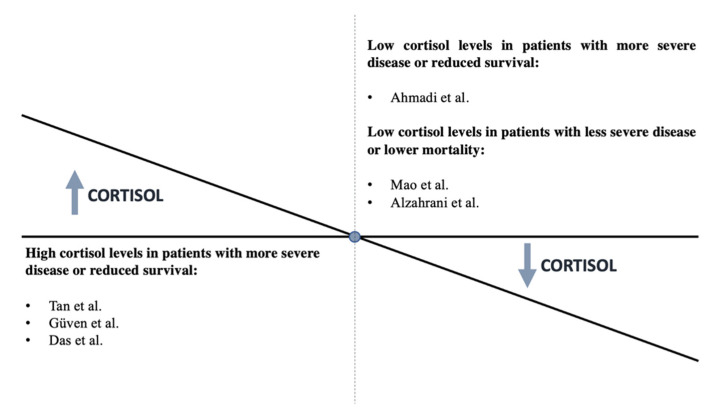
Association between cortisol levels and disease severity [17,18,66,67,68,69].

By contrast, the most recent findings revealed that individuals with SARS-CoV-2 who had lower cortisol levels had a greater fatality rate [66]. A total of 154 hospitalized patients with COVID-19 were studied in a prospective cohort study. ACTH and cortisol levels in the blood were measured on the first or second day of hospitalization. Cortisol levels were substantially lower in those who died (311.7 (273.1–394.5) nmol/L) than in patients who were discharged (460.7 (344.8–598.6) nmol/L) (*p* = 0.003), while ACTH levels were unaffected. According to the logistic model, cortisol levels that rose by one unit correlated with a 26% lower mortality risk [66]. Overall, the existing evidence does not consistently support an association of severe COVID-19 with the presence of reduced or increased cortisol. Figure 2 summarizes the studies to date regarding the association between cortisol levels and disease severity. Further precise investigations with long-term follow-up are necessary.

### 4.7. Clinical Data in Patients Who Survive COVID-19 Infection

It is increasingly evident that the health impact of COVID-19 extends beyond the initial infection, with up to 63% of patients reporting ongoing symptoms [70]. Fatigue and some other symptoms could overlap with “post-COVID” or “long COVID” and AI. As AI is eminently treatable, it is imperative to identify any contribution it may have to the persistent symptoms experienced by patients after COVID-19 infection. A recently published study assessed the HPA axis in patients at least 3 months after diagnosis of COVID-19. They all had peak cortisol ≥450 nmol/L after tetracosactide, Synacthen (250 micrograms iv bolus), consistent with adequate adrenal reserve. Basal and peak serum cortisol did not differ according to disease severity or history of dexamethasone treatment (6 mg once daily for a maximum of 10 days during COVID-19). Patients who were prescribed other steroids (oral, inhaled, topical, or intra-articular) following recovery from COVID-19 and those taking other medications known to affect CBG (including oral estrogens) were excluded from the study. The authors reported for the first time that the fatigue after COVID-19 was not accounted for the overt adrenal dysfunction [70].

Another group analysed the persistence of symptoms and their impact on quality of life in people who had had COVID-19 one year after their admission for COVID-19, and they explored the influence of treatment with systemic corticosteroids during the acute phase of the illness. Most symptoms were less frequent in the group that received corticosteroids, with statistically significant differences for headache, dysphagia, chest pain, and depression. These patients also showed significantly better outcomes in the SF-36 domains for “bodily pain” and “mental health.” They concluded that corticosteroids administered in the acute phase of COVID-19 could attenuate the presence of long-term symptoms and improve patients’ quality of life [71].

## 5. Pre-Existing HPA Axis Disorders and COVID-19 Outcomes

**Figure 3:**
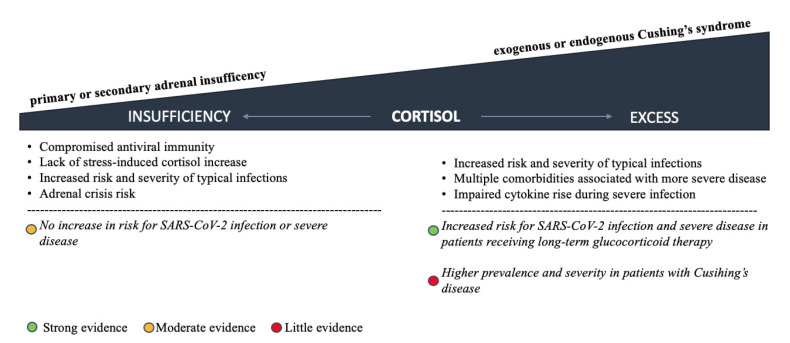
Glucocorticoid disorders and their impact on risk of infection and disease severity.

The high degree of endocrine system involvement in COVID-19 quickly indicated the clinical relevance of pre-existing endocrine disorders [1]. Both impaired cortisol secretion and excess, as observed with AI and Cushing’s syndrome, may increase susceptibility to SARS-CoV-2 infection and the risk of severe forms of COVID-19 and therefore position those patients in potentially vulnerable groups [72]. As well as defined adrenal disorders, long-term glucocorticoid treatment should be considered the most common cause of adrenal dysfunction in the general population [73]. In addition, glucocorticoid therapy for moderate to severe COVID-19 cases may either cause Cushing’s syndrome at supraphysiologic doses or AI because of HPA axis suppression after drug withdrawal and consequently affect COVID-19 outcomes in any direction [73]. The spectrum of pre-existing glucocorticoid disorders and their impact on infection risk and COVID-19 severity is presented in Figure 3.

### 5.1. Adrenal Insufficiency

Patients with AI possess compromised antiviral immune defense mechanisms due to the impaired innate immune system with defective activity of neutrophils and natural killer cells [74,75]. In addition, the lack of a physiological stress-induced increase in serum cortisol during infection might further predispose them to progression to more severe disease stages [76]. Previous studies reported an increased risk of infections and increased hospitalization and mortality rates in AI patients compared to controls [76,77,78]. An additional concern is a high risk of an adrenal crisis during severe infections [79]. Even when AI patients receive optimal treatment, the morbidity and mortality rates continue to be higher than in the general population [73]. A recent paper demonstrated that patients on conventional glucocorticoid therapy had a pro-inflammatory state and weakened immune response, and a normalization of their immune system was observed with the restoration of the physiological circadian cortisol rhythm by modified-release hydrocortisone [80].

Information regarding the prevalence and severity of COVID-19 in AI patients is scarce, making the assessment of their vulnerability difficult. During the first wave of the pandemic, three cross-sectional studies reported a low prevalence of SARS-CoV-2 infections among AI patients, with the disease severity similar to healthy controls [81]. In a retrospective case–control study, including 279 patients with primary and secondary AI and 112 controls, the prevalence of symptomatic patients was similar between cases and controls, no AI patient required hospitalization, and no adrenal crisis was reported [82]. The second study included 121 AI patients and demonstrated a lower prevalence of COVID-19 in that group compared to healthy controls and no need for hospitalization [83]. The last study again noted low prevalence and no need for hospitalization or reported adrenal crisis in 159 patients with secondary AI [84]. A more recent longitudinal survey conducted in two tertiary medical centers in the US confirmed these findings with a significantly lower prevalence of COVID-19 among AI patients compared to the overall population (1.8% vs. 7.9%) [85]. Therefore, based on all the reported data so far, there is no evidence to suggest that patients with primary or secondary AI are at increased risk of SARS-CoV-2 infection or a severe COVID-19 course if they are adequately educated about procedures during infection [75,81].

In order to accommodate for the insufficient serum cortisol increase during infection, AI patients are advised to follow the “sick day rules” immediately when the first symptoms might appear by a self-adjusted rise in hydrocortisone dose administration. They are encouraged to contact their referral endocrinologist and continue with doubled or tripled daily oral glucocorticoid doses in relation to the severity of COVID-19 symptoms until the symptoms resolve [1,72,74,81]. Hospital admission and treatment with parenteral glucocorticoids are recommended in the case of clinical deterioration or if the patients experience vomiting or diarrhea [74,86]. Regarding the management of substitutive therapy in patients with AI who have received a COVID-19 vaccine, a recent survey by the Pituitary Society reported that 36% of clinicians recommended an increased glucocorticoid dosage after the first injection. In contrast others planned to increase replacement therapy only in the case of fever or other vaccination-related symptoms [87].

### 5.2. Glucocorticoid Excess

Hypercortisolemia induces persistent low-grade inflammation and immunosuppression with a hampered innate immune response and lowered activation of adaptive immune mechanisms. Indeed, patients with exogenous or endogenous glucocorticoid excess have increased susceptibility to infections and frequently experience a more severe and protracted clinical course of respiratory viral infections and other various fungal, bacterial, and viral infections [72,88]. In addition, multiple comorbidities known to increase COVID-19 severity such as impaired glucose metabolism, arterial hypertension, obesity, and hypercoagulability are overrepresented in these patients and commonly persist despite the remission of the primary disease [88,89]. However, since these patients might have impaired cytokine rise during severe infection, they might paradoxically be less prone to progressing to ARDS in COVID-19 [72,76,90]. Furthermore, the initial signs and symptoms of COVID-19 in this group of patients might be misleading, with typical symptoms such as fever and dyspnea frequently absent [20].

The higher prevalence makes the data regarding exogenous glucocorticoid excess more robust; however, despite the best efforts, it is difficult to definitively conclude the vulnerability to SARS-CoV-2 infection in these patients. The growing amount of evidence suggests a higher risk of infection and severe disease course in patients receiving high-dose glucocorticoid therapy for autoinflammatory conditions after contracting SARS-CoV-2 [20]. Meta-analyses predominantly observed associations between glucocorticoid treatment and adverse COVID-19 outcomes and demonstrated a particularly high risk in patients with high-dose glucocorticoid therapy [20]. On the other hand, less data are available to analyse the risk for patients with endogenous Cushing’s syndrome [20]. The first published study by a single tertiary centre in Lombardy on a small number of Cushing’s disease patients during the first wave of the pandemic demonstrated a higher prevalence of COVID-19 compared to the general population (3.2% vs. 0.6%) and severe clinical presentation was particularly reported in patients with active disease [91]. Following that study, only a few cases of patients suffering from endogenous hypercortisolemia and COVID-19 have been reported to date [89]. The clinical course of COVID-19 in published cases ranged from mild to death, and it seems that the severity of COVID-19 in that group of patients might depend on the intensity of endogenous hypercortisolism [91,92,93,94]. 

Since patients treated with supraphysiological glucocorticoid doses might be at increased risk of severe COVID-19 due to associated metabolic and cardiometabolic complications, using the lowest effective glucocorticoid dose is recommended if the underlying disease is well controlled [20,95]. Abrupt discontinuation of glucocorticoid treatment should be avoided to prevent HPA axis suppression. Patients with active Cushing’s disease infected with SARS-CoV-2 might be best treated with a “block and replace” approach to reduce the risk of AI crises caused by an impaired HPA axis dynamic [72]. Highly prevalent comorbidities such as diabetes and hypertension should be appropriately managed, as they present significant risk factors for adverse COVID-19 outcomes [76]. Hypercortisolemia, acute inflammation, and immobility are well-documented factors promoting clot formation, and therefore antithrombotic prophylaxis should be introduced early in the disease process [89]. Furthermore, patients with active Cushing’s syndrome are at increased risk for prolonged viral shedding and opportunistic infections; therefore, prolonged antiviral treatment and empirical prophylaxis with broad-spectrum antibiotics should be considered for hospitalized patients [89,90].

## 6. Conclusions

This review was conducted to present an endocrinological point of view on the relationship between SARS-CoV-2 infection, its direct HPA axis effects, the severity status of the disease, and the whole spectrum of the potential COVID-19-related HPA axis dysfunction, from endogenous- and exogenous-induced glucocorticoid insufficiency to excess. Despite limited and inconsistent clinical data, the current literature supports multiple mechanisms that can cause HPA axis impairment beyond GI-AI. The current level of evidence enables some general recommendations for researchers and clinicians managing COVID-19 patients worldwide. Since SARS-CoV-2, directly and indirectly, affects the HPA axis, AI is a probable and mostly unrecognized condition. In addition, glucocorticoids remain the primary therapy for moderately and severely ill patients; thus, in all patients, a low threshold to test for AI is needed, even more importantly after glucocorticoid withdrawal. This is particularly important for patients with nonspecific symptoms after the infection or glucocorticoid cessation, including those with symptoms attributed to long COVID. 

In addition, patients with pre-existing HPA axis disorders should still be considered potentially vulnerable groups. However, low-quality evidence suggests that patients with primary or secondary AI are not at increased risk of SARS-CoV-2 infection or more severe disease course if they are adequately educated about procedures during infection. On the other hand, some evidence supports a higher prevalence and severity in patients with Cushing’s syndrome, and even stronger data support an increased risk for SARS-CoV-2 infection and severe disease in patients receiving long-term glucocorticoid therapy.
